# Aggregate Geometrical Features and Their Influence on the Surface Properties of Asphalt Pavement

**DOI:** 10.3390/ma15093222

**Published:** 2022-04-29

**Authors:** Pei Sun, Ke Zhang, Sen Han, Yun Xiao

**Affiliations:** 1 School of Urban Construction and Transportation, Hefei University, Hefei 230601, China; xiaoyun@hfuu.edu.cn; 2 College of Information Engineering, Fuyang Normal University, Fuyang 236041, China; zhangke_365@126.com; 3 Highway and Airport Pavement Research Center, School of Highway, Chang’an University, Xi’an 710064, China; hyram_hs@chd.edu.cn; 4 Anhui Province Transportation Big Data Analysis and Application Engineering Laboratory, Hefei University, Hefei 230601, China

**Keywords:** asphalt mixture, aggregate geometrical features, digital image processing, pavement surface properties, walking friction tester

## Abstract

Aggregate geometrical features directly affect asphalt pavement surface properties, which further affect the resistance to skidding of a road surface. In order to establish a relationship between the aggregate geometrical features and pavement surface properties, this paper employs an aggregate geometric characteristic evaluation system (AGCES) to describe the form property, angularity, and surface texture of aggregate particles. The geometrical feature parameters of 15 different aggregates were examined by AGCES and the corresponding surface properties of asphalt pavement prepared from the mentioned aggregates were evaluated by sand patch method, 2-Dimension Image-based Texture Analysis Method (2D-ITAM) and Walking Friction Tester (WFT), respectively. The relationships between the pavement surface property parameters and the aggregate geometric characteristic parameters studied were developed by the Levenberg-Marquarat and universal global optimization (LM-UGO). The results show that the calculated geometric characteristic parameters are in agreement with traditional manual measurement results. The pavement surface properties are significantly influenced by aggregate angularity and aggregate surface texture. Regression relationships were established to predict pavement surface properties from the aggregate geometrical features.

## 1. Introduction

As the largest component of asphalt mixture, the properties of aggregate particles directly affect the asphalt pavement performance [1,2]. Previous studies have shown that the geometrical features of aggregates (i.e., shape, angularity and surface texture) affected the degree of interlocking of aggregate particles and the interaction between aggregate particles and asphalt binder during compaction, thus directly affecting the pavement performance of the mixture [3,4,5,6].

There are two types of methods to obtain aggregate geometrical characteristics: traditional manual measurement methods and digital image processing techniques [7,8,9]. The traditional testing methods are time-consuming and can easily be affected by subjective factors. Therefore, in the design of asphalt mixture, the requirements for aggregate particles focus on the frictional resistance and wheel-polishing resistance, without involving the aggregate geometric characteristics. In recent years, with the development of digital image processing (i.e., DIP) techniques, many scholars use DIP techniques to explore the geometric characteristics of aggregate. Among them, the University of Illinois Aggregate Image Analyzed (UIAIA) system and the aggregate image-measurement system (AIMS) are widely accepted and used [10,11,12]. The above two systems can be used to evaluate the geometric characteristics of aggregates accurately, but all need advanced equipment.

Based on the digital image processing technique, many scholars tried to explore the relationship between geometric characteristics of aggregate and pavement performance of asphalt mixture. Topal et al. proposed that the mineral properties and crushing method of fine aggregate must be considered in the study of fine aggregate angularity property, and the asphalt pavement prepared by fine aggregate with more prominent edges and angles had better resistance to rutting [13]. Rousan et al. pointed out that during the compaction stage, the geometric properties of aggregate directly affected the intercalation between aggregate particles and the cohesive force between aggregate particles and asphalt binder, which further affected the road performance of the asphalt mixture [14]. The results of the study by Bessa et al. showed that the asphalt mixtures prepared by aggregates with different mineralogical properties but similar shape properties may result in similar mechanical performances [15]. Pei et al. proposed that the rheological properties of the mixture during the compaction and service process were sensitive to the sieve diameter and shape properties of the aggregate, while the angularity properties of the aggregate had no significant effect on the rheological properties of the mixture [16]. Hassan et al. analyzed the internal structure of asphalt mixture prepared by three aggregates with different shape properties, and tried to establish the relationship between the internal structure of asphalt mixture and pavement performance. Results show that the shape and strength of aggregate have a significant effect on the internal structure of asphalt mixture, and then affect the high-temperature and low-temperature performance of asphalt pavements [17].

Although great achievements have been made in the research on aggregate geometrical features and pavement performance, the way in which the aggregate geometrical features affect pavement surface properties is an area that has not received much attention. Pavement surface properties, especially surface texture characteristics, directly affect skid resistance and noise reduction performance of pavements [18,19,20]. Establishing the relationship between the aggregate geometrical features and the pavement surface properties will help us to understand the development of pavement anti-sliding noise reduction better.

The primary objective of this study is to explore the correlation between pavement surface properties and aggregate geometrical features. The pavement surface property parameters are represented by mean texture depth (MTD), mean profile depth (MPD) and WFT friction coefficient (WFC), while aggregate geometrical features are characterized by Shape index (SI), form factor (FF), angularity index (AI) and texture factor (TF). Attempts are made to develop prediction models of surface properties of pavements from aggregate geometric characteristic parameters.

## 2. Experimental Study

### 2.1. Raw Materials and Mixture Design

#### 2.1.1. Raw Materials

Previous studies showed that the influence of coarse aggregate on the pavement surface property is much greater than that of fine aggregate [21,22,23], so only the coarse aggregate particles (i.e., particle size > 2.36 mm) were considered in this study of the aggregate geometrical features. Limestone, round limestone, basalt, diabase and gneiss with different geometric characteristics were selected to study the influence of aggregates on the pavement surface properties. The round limestone was obtained by limestone with the Los Angeles abrasion tester. The Los Angeles abrasion tester is manufactured by Zhong Ke Road Construction Co., Ltd., Beijing, China. The test program is shown in Table 1.

The research object is the overall shape properties of aggregates with different particle sizes and the shape of the final projection surface of the aggregate is affected by the placement position. However, the two-dimensional shape of the aggregate is only the shape of a projection surface, which may not represent the characteristics of the whole aggregate; therefore a large sample number was selected to reduce error. The total number of aggregate samples of each type selected in this study was 160.

#### 2.1.2. Mixture Design

Dense graded asphalt gradation AC-13 coarse, AC-13 target and AC-13 fine were used in this study, as shown in Table 2. SBS modified asphalt was selected to prepare mixture specimens, and the optimum asphalt-aggregate ratio is 4.7%, 4.8% and 4.9%, respectively.

Specimens having dimensions of 50 cm × 50 cm × 5 cm were prepared. Mixture samples were compacted in a laboratory using a hand-held roller compactor. The compaction times are 24 round trips and the compaction temperature is guaranteed to be 175 °C.

### 2.2. Aggregate Geometrical Features Analysis Method

#### 2.2.1. Image Processing

**Figure 1:**
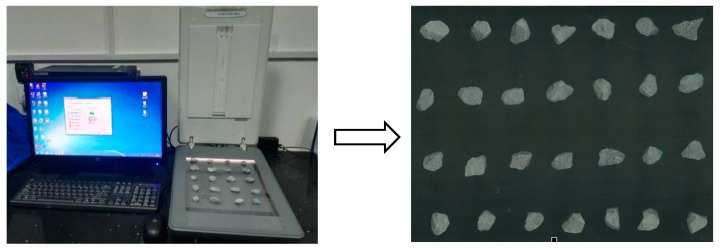
Aggregate particle image acquisition.

The sieved aggregate particle image (i.e., RGB image) was obtained by a scanner at a resolution of 1200 pix/inch, as shown in Figure 1. Firstly, the RGB image was converted to a grey image. Secondly, the median filter was applied to eliminate the random noise caused by the scanner. Then, the image was binarized. After binarization, the image was processed by an opening-and-closing operation, in which the open operation was to eliminate isolated pixels of the aggregate edges, and the closed operation was to eliminate black spots in the aggregate. Finally, the aggregate was marked.

#### 2.2.2. Aggregate Geometric Features Analysis

Generally, the geometrical features of aggregate particles can be described by three independent feature components: form, angularity, and surface texture [24].

Three indicators, namely shape index (i.e., *SI*), shape factor (i.e., *SF*), and form factor (i.e., *FF*), were used to evaluate the form properties of the aggregate particle, as shown in Equations (1)–(3).

*SI* and *SF* were calculated by equivalent the aggregate particle into an ellipse, which had the same area and the same first and second moments as the target area. The value of *SI* is greater than or equal to 1, and the closer *SI* is to 1, the closer the particle is to a regular polygon or circle. The value of *SF* is 0–1. With the increase of *SF*, the aggregate particles become more and more slender. The value of *FF* is not greater than 1, and the closer *FF* is to 1, the closer the aggregate particle is to the circle.

(1) $$SI={\left(L_{\max}/L_{\min}\right)}^{2}$$

(2) $$SF={\left(c/L_{\max}\right)}^{2}$$

(3) $$FF=\frac{4\pi A}{l^{2}}$$

where, *L*_max_ and *L*_min_ represent the length of the long axis and the short axis of the equivalent ellipse, respectively, *c* is the focal length of the equivalent ellipse, *A* and *l* represent the area and perimeter of the aggregate particle in the image, respectively.

Angularity index (i.e., *AI*) calculated according to Equation (4) was used to evaluate the aggregate angularity. The more significant the edges and corners of the aggregate, the higher the *AI*.

(4) $$AI={\left(P/P_{e}\right)}^{2}$$

where *P* is the perimeter of the aggregate particle in the image and *P_e_* is the perimeter of the equivalent ellipse.

Aggregate surface texture was collected by using the aggregate area change scale under high resolution. The texture factor (i.e., *TF*) was determined according to Equation (5). With the increase of *TF*, the surface texture of aggregate is more abundant.

(5) $$TF=\frac{A_{1}-A_{2}}{A_{1}}\times 100$$

where, *A*_1_ is the area of the aggregate in the image, *A*_2_ is the area of the aggregate obtained after the application of “corrosion-expansion” technology in the image.

**Figure 2:**
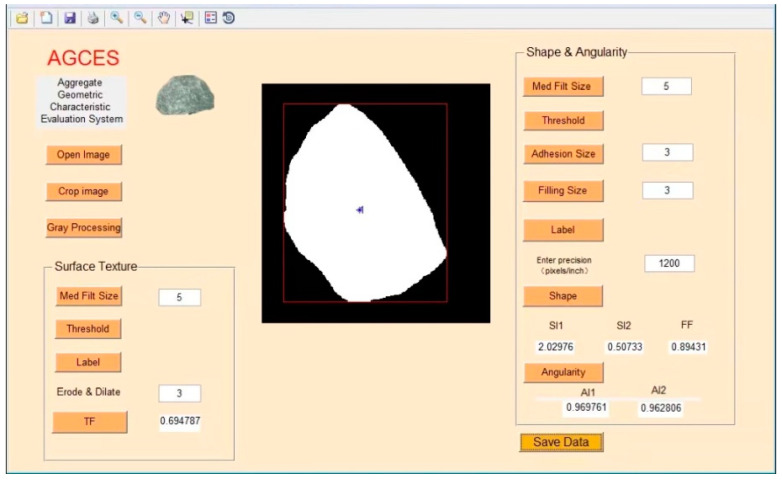
AGCES software interface.

For convenience, the whole image process can be performed using the Aggregate Geometric Characteristic Evaluation System (i.e., AGCES) which was developed in this study based on Matlab, as shown in Figure 2.

### 2.3. Pavement Surface Property Analysis Method

The mean texture depth (MTD) and mean profile depth (MPD) measured by the sand patch method and 2-Dimension Image-based Texture Analysis Method (2D-ITAM) were used in the study to evaluate the pavement surface macro-texture [25,26]. The WFT friction coefficient (WFC) measured by the Walking Friction Tester (WFT) was used in the study to evaluate the pavement surface micro-texture.

The 2-Dimension Image-based Texture Analysis Method firstly obtains the surface profile from the asphalt mixture section image based on digital image processing techniques, and calculates the mean of elevation of all data points on the surface profile after pretreatment, which is denoted as *Z_ave_*. Then, the surface profile is divided into two sections along the midpoint, and the maximum elevation points of the former and the latter are found respectively, which are denoted as *Z*_max1_ and *Z*_max2_. MPD is calculated according to Equation (6).

(6) $$MPD=\frac{Z_{\max}{}_{1}+Z_{\max}{}_{2}}{2}-Z_{ave}$$

**Figure 3:**
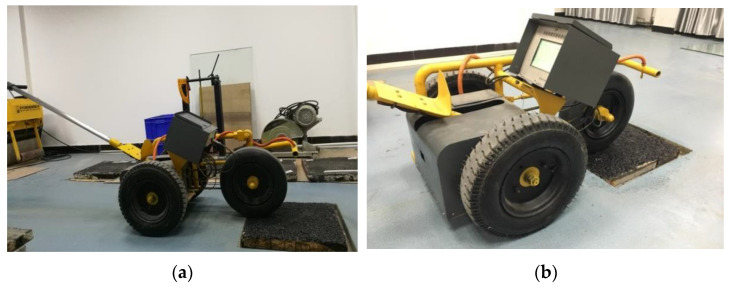
The Walking Friction Tester. (**a**) Test wheel contact the specimen; (**b**) Test procedure.

The Walking Friction Tester developed by Chang’an University (Xi’an, China) can be used to measure the low-speed pavement friction, which avoids the disadvantages of BPT and DFT, such as the mode of friction testing and the shape of their sliders [27], as shown in Figure 3. The WFT has three wheels with a diameter of 400 mm and the front wheel is the test wheel. The test wheel is a solid smooth rubber tire with a constant vertical load of 196 N. The contact pressure is about 99.2 KPa. The torque sensor installed on the front axle and the speed sensor installed on the rear axle transmit the torque signal and speed signal respectively to the automatic data acquisition instrument, which simultaneously stores the current test speed.

The WFT can switch five slip ratios, i.e., 0%, 10%, 20%, 30% and 100%, and the test speed range is allowed from 10 m/min to 100 m/min. The experiment shows that the test speed has little influence on the test results and the friction coefficient is more sensitive to the micro-texture when the test speed is lower, so the speed of 15 m/min was adopted in this study. Meanwhile, the WFT is equipped with a water container to ensure a surface water film thickness of 0.3 mm during testing. WFC is calculated according to Equation (7).

(7) $$WFC=\frac{M}{R\times N}$$

(8) $$\mu =\frac{F}{N}$$

where *M* and *μ* are torque and friction coefficients measured by WFT respectively, *R* is the radius of the test wheel, *N* is the vertical load of the test wheel, and *F* is the friction of the tire-road contact surface.

**Figure 4:**
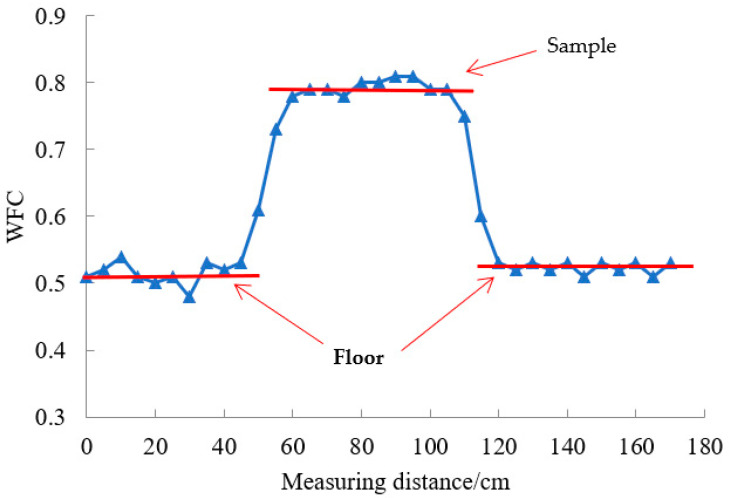
Sensitivity analysis of WFT to micro-texture.

When the WFT is running on a smooth floor, the WFT friction coefficient is small. When the test wheel just touches the sample, the WFT friction coefficient will suddenly increase, and when the WFT moves away from the sample, the WFT friction coefficient will decrease again, as shown in Figure 4.

## 3. Aggregate Geometrical Features Test Results

### 3.1. Analysis of Aggregate Form

**Figure 5:**
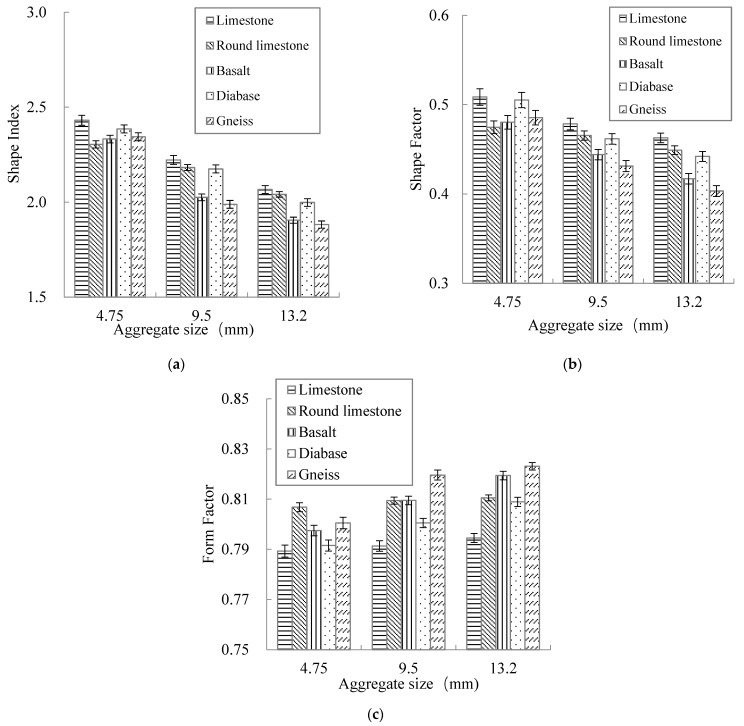
Test results of aggregate form properties: (**a**) Shape index, (**b**) Shape factor, (**c**) Form factor.

The form properties of 15 kinds of broken aggregates were tested by the AGCES, as shown in Figure 5.

Figure 5 shows that with the increase of aggregate particle size, the shape index and shape factor decreased gradually, and the form factor got closer to 1, indicating that the aggregate has lower flat and elongated particle values and becomes more circular in two dimensions. Compared with limestone, the shape index and shape factor of round limestone both decreased, and the form factor gradually approached 1, which showed that the abrasion changed the overall shape of aggregate particles and made them smooth.

For aggregates with the same particle size (except for round limestone), basalt and gneiss were rounder, while the limestone and diabase had higher flat and elongated particle values. But on average, all four aggregates showed little difference in form characteristics and the form properties were not significantly related to the aggregate mineralogy. This is because compared with the mineralogical property of aggregate, the production processes had a more significant impact on the form properties of aggregate. Because the four types of aggregates were produced using similar processes in the quarry, the difference in form properties was not obvious.

### 3.2. Analysis of Aggregate Angularity

**Figure 6:**
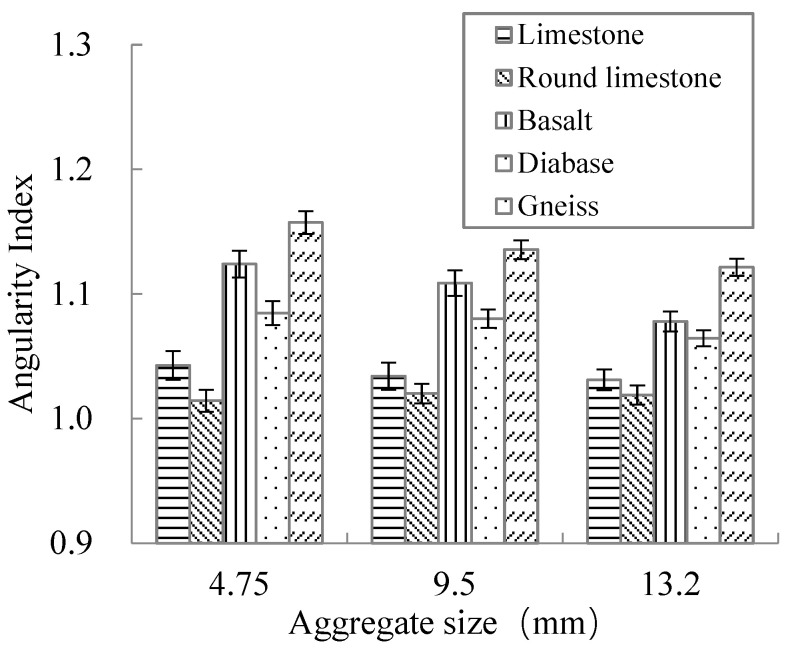
Test results of aggregate angularity index.

The angularity results of 15 kinds of broken aggregates are shown in Figure 6.

Figure 6 shows that as the aggregate size increased, the angularity index gradually decreased. That is, the edges and corners of aggregate particles became less conspicuous. Compared with limestone, the edges and corners of round limestone were gradually polished to smooth.

Meanwhile, for aggregates with the same particle size (except for round limestone), gneiss had the most prominent edges and corners, followed by basalt and diabase, while limestone had the least. This is mainly due to the difference in the mineral chemical composition of different mineralogical aggregates, which is manifested as the difference in physical and mechanical properties. The main component of limestone is calcite. Due to the low strength of calcite, the edges and corners of limestone are easily “flattened” during the production processes in the quarry, resulting in the edges and corners of aggregate not being obvious enough. The basalt surface is mostly banded, and the gneiss surface also has an obviously banded structure, which makes them have more prominent edges and corners.

### 3.3. Analysis of Aggregate Surface Texture

**Figure 7:**
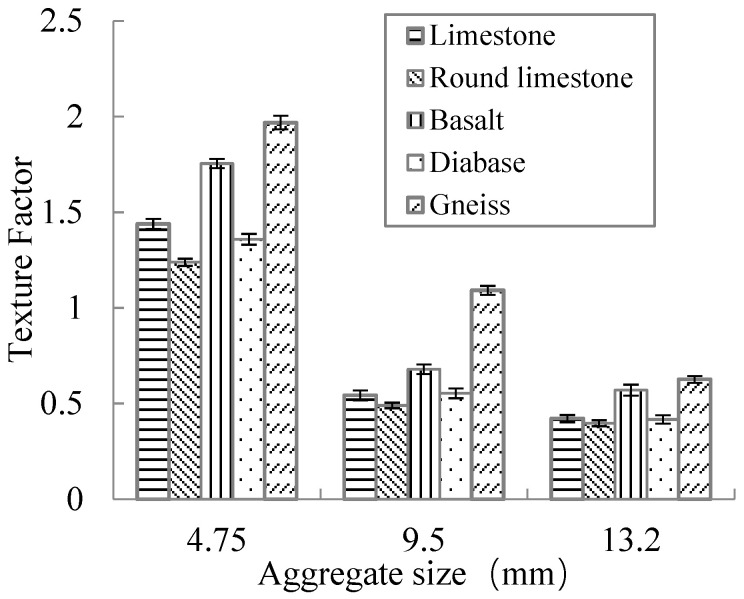
Test results of aggregate surface texture indicator.

The texture factor results of aggregates are shown in Figure 7.

As can be seen in Figure 7, the texture factor of limestone decreased slightly after the abrasion. With the increase in aggregate particle size, the texture factor decreased, indicating that the surface texture of aggregate particles decreased.

Figure 7 also shows that for aggregates with the same particle size (except for round limestone), the texture factor always meets the following requirements: gneiss > basalt > limestone > diabase > round limestone. This is due to the difference in aggregate surface texture coming from the difference in its original rock section structure, while the original rock section structure mainly depends on the mineralogy and diagenetic mechanism of aggregate.

### 3.4. Experimental Verification

In order to select the most appropriate evaluation index of aggregate form properties and verify the accuracy of using digital image processing techniques to obtain the evaluation indices of aggregate geometrical features, the results of aggregate geometric characteristic parameters calculated in Section 3.1, Section 3.2 and Section 3.3 were further verified.

#### 3.4.1. Selection of Aggregate Form Characteristic Index

**Figure 8:**
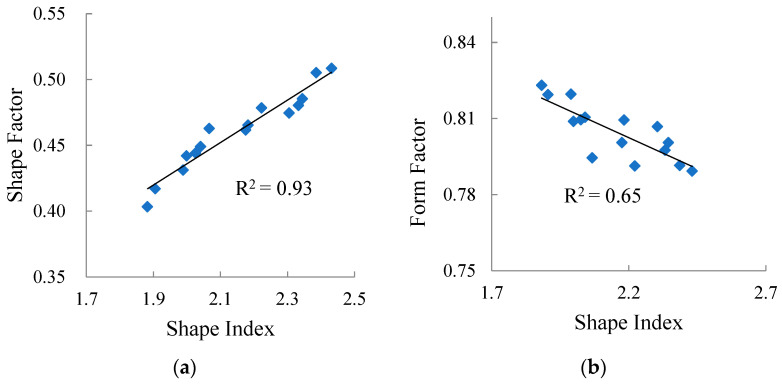

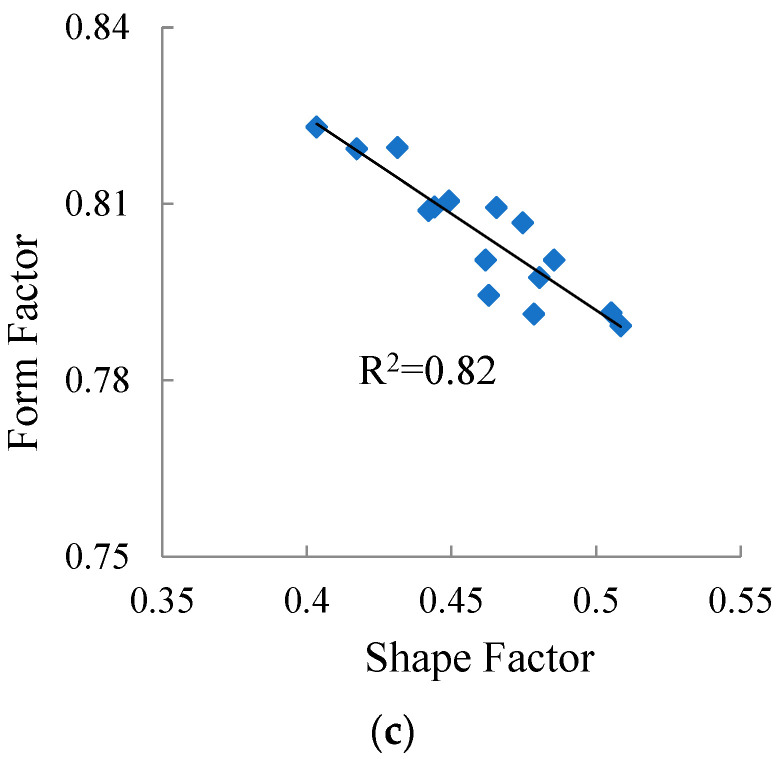
Correlation between aggregate form evaluation indices: (**a**) Shape index and shape factor, (**b**) Shape index and form factor, (**c**) Shape factor and form factor.

Among the selected evaluation indices of aggregate form properties, there may be overlap of aggregate form information, so the correlations between the three indices (i.e., *SI*, *SF* and *FF*) were analyzed, and the results are shown in Figure 8.

It can be seen from Figure 8 that there is a strong correlation between shape index and shape factor, and a good correlation between shape factor and form factor, indicating that there is partial overlap between shape factor and two other evaluation indicators.

In addition, the correlation coefficient between shape index and form factor is only 0.65, much weaker than the correlation coefficient between shape index and shape factor, and between shape factor and form factor. Therefore, in subsequent studies, only shape index and form factor were used to evaluate the form properties of aggregate.

#### 3.4.2. Verification of Aggregate Angularity Characteristic Index

**Figure 9:**
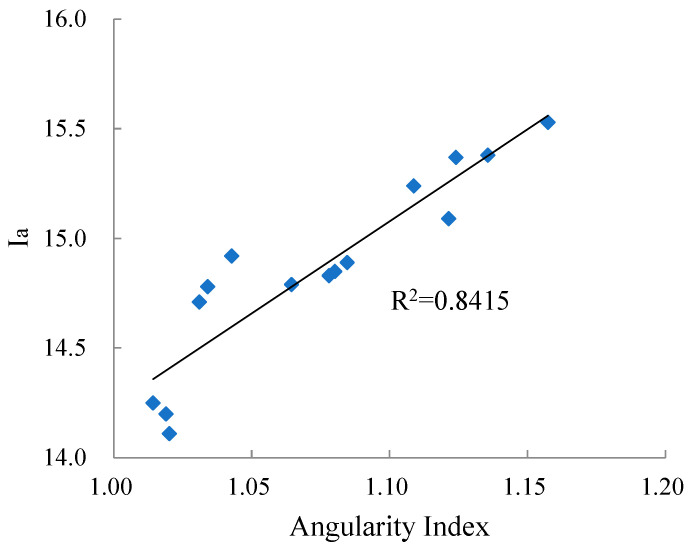
Correlation between aggregate angularity index and Ia.

ASTM D3398 method was used to determine the aggregate angularity, and the evaluation index was Ia [28]. Correlation of Ia measured by ASTM D3398 and angularity index (i.e., *AI*) calculated by the AGCES are shown in Figure 9.

According to Figure 9, the correlation coefficient between Ia and angularity index is 0.8415, indicating that there is a strong correlation between the two. This shows that aggregate angularity parameters calculated by AGCES can be used to estimate the aggregate angularity.

#### 3.4.3. Verification of Aggregate Surface Texture Characteristic Index

In order to verify the feasibility of texture factors (i.e., *TF*), a single factor analysis method was used to test whether the index could reflect the surface texture differences of aggregates with different mineralogical properties and different particle sizes.

The 15 types of aggregates were divided into two groups for One-Way ANOVA. One group was aggregates with the same mineralogical property and different particle sizes, while the other group was aggregates with different mineralogical properties and same particle size. The test results are shown in Table 3 and Table 4.

Table 3 and Table 4 show that the F values of test statistics are much higher than F_0.05_ (2477) and F_0.05_ (4795), indicating that under the significant level of 0.05, it is believed that the texture factor can significantly represent the surface texture differences of aggregates with different mineralogical properties and different particle sizes.

## 4. Statistical Relationships between Pavement Skid Resistance and Aggregate Geometric Features

In this section, the statistical relationships between the aggregate geometric characteristic parameters described in the preceding section and selected evaluation parameters of pavement skid resistance are examined. The evaluation parameters of pavement skid resistance selected were MTD, MPD and WFC (see Section 2.3).

### 4.1. Results of Aggregate Geometrical Features in the Mixture

Taking limestone and AC-13 target as an example, the average SI, average FF, average AI and average TF of aggregates in the asphalt mixture were calculated using the method in Table 5. The results of aggregates features in the mixture are shown in Table 6.

Table 6 shows that the average SI of aggregates in the mixture (i.e., SIa) and the average FF of aggregates in the mixture (i.e., FFa) have a small difference in values. The average AI of aggregates in the mixture (i.e., AIa) satisfies: gneiss > basalt > diabase > limestone > round limestone, while the order of the average TF of aggregates in the mixture (i.e., TFa) is: gneiss > basalt > limestone > diabase > round limestone.

### 4.2. Statistical Correlations of MTD and MPD with Aggregate Geometrical Features

**Figure 10:**
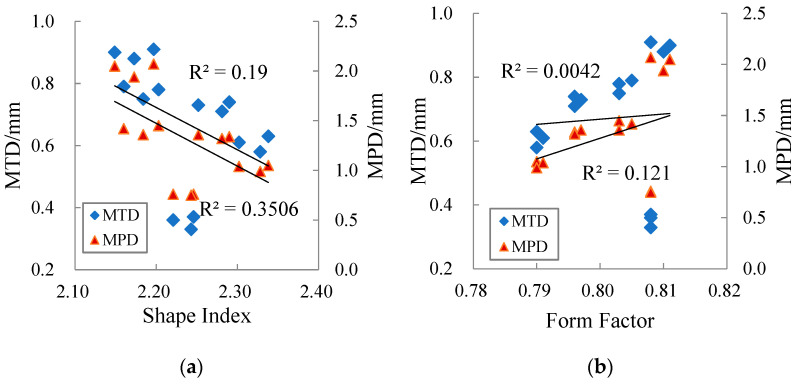

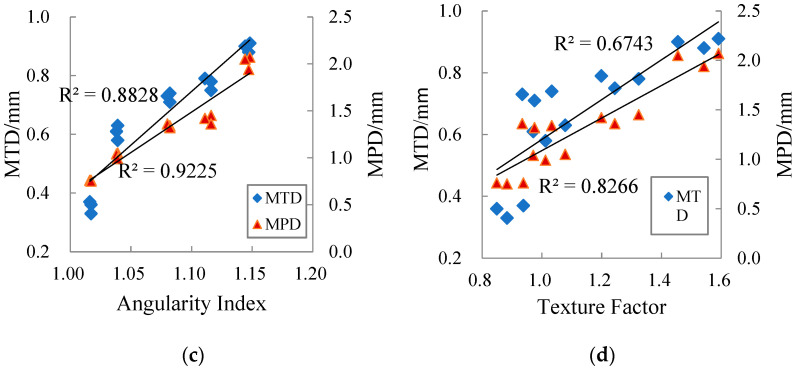
Correlation between MTD/ MPD and four aggregate geometric characteristic parameters: (**a**) Average shape index, (**b**) Average form factor, (**c**) Average angularity index, (**d**) Average texture factor.

The correlations of MTD and MPD with the aggregate geometric characteristic parameters are shown in Figure 10.

Figure 10 shows that MTD and MPD are positively correlated with the average angularity index and the average texture factor. This is because the angularity property and surface texture of aggregate affect the degree of interlock of aggregate particles and the interaction between aggregate particles and asphalt binder. Aggregate with no abundant edges or corners and no obvious surface texture is more easily to be compacted to a dense state during the process, thus reducing the volume of air voids and making the surface macro-texture worse.

Figure 10 also shows that the correlations of MTD and MPD with the average shape index and the average form factor are poor. The shape index mainly describes the degree of flatness and elongation of aggregate, and the form factor describes the roundness of aggregate. Aggregates that have higher flat and elongated particle values (i.e., higher shape index) in mixture are easy to break during compaction and have the tendency to reduce the degree of interlock of aggregate particles, while aggregates inclined to round in the mixture are favorable to the compaction process. However, there is no rule that “the form factor of aggregate with large shape index must be small”.

Generally speaking, the smaller the shape index and the larger the form factor in the aggregate particles, the worse the corresponding pavement surface macro-texture. This is mainly because compared to the point-to-point contact between flat and elongated aggregates, the contact form between spherical particles is closer to face-to-surface contact, and is easier to be compacted to a dense state, which results in a decrease in MTD and MPD.

### 4.3. Statistical Correlations of WFC with Aggregate Geometric Features

**Figure 11:**
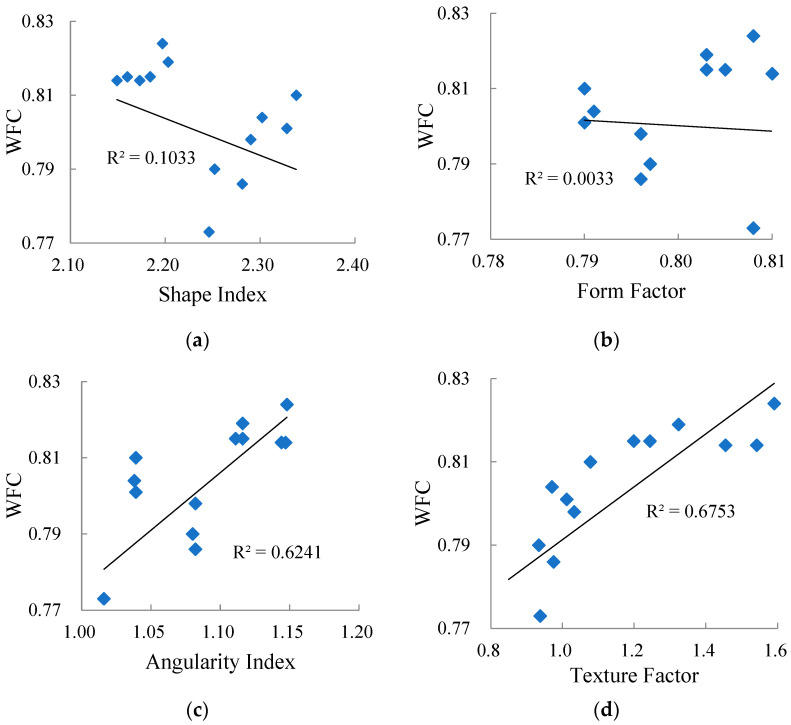
Correlation between WFC and four aggregate geometric characteristic parameters: (**a**) Average shape index, (**b**) Average form factor, (**c**) Average angularity index, (**d**) Average texture factor.

The correlation of WFC with the aggregate geometric characteristic parameters is shown in Figure 11.

From Figure 11, it can be seen that the correlation between WFC and average shape index/average form factor is poor; that is, the influence of aggregate form properties on WFC is not exactly regular. Figure 10 also shows that with the increase of average angularity index and average texture factor, WFC increased. This is because the aggregate particles with poor angularity and a smooth surface have difficulty puncturing the water film, resulting in the water not being discharged smoothly, whereas the tire and the road cannot be completely in contact. Moreover, the water film plays a lubrication role, which leads to a decrease in friction coefficient.

## 5. Prediction Model of Pavement Surface Properties Based on Aggregate Geometrical Features

Levenberg-Marquart and universal global optimization (i.e., LM-UGO) have a strong ability of optimization and fault tolerance. They solve the problem that initial parameter values must be given in the process of fitting by calculating the optimal solution through their unique global optimization algorithm. Considering the advantages, LM-UGO was used to calculate the parameters of the regression models.

In order to establish the prediction model of pavement surface properties based on the aggregate geometric characteristic parameters, the average angularity index (i.e., *AI*a) and the average texture factor (i.e., *TFa*) were taken as independent variables, while *MTD*, *MPD* and *WFC* were taken as dependent variables. The statistical regression prediction models were established as follows.

(9) $$MTD=4.1531AI_{a}-0.1121TF_{a}-3.6846{\;R}^{2}=0.89$$

(10) $$MPD=6.7514AI_{a}+0.4139TF_{a}-6.4471{\;R}^{2}=0.93$$

(11) $$WFC=0.1001AI_{a}+0.0453TF_{a}+0.6405{\;R}^{2}=0.69$$

The three regression models above were tested by the F-tests and were highly significant statistically at a significance level of 95%.

**Figure 12:**
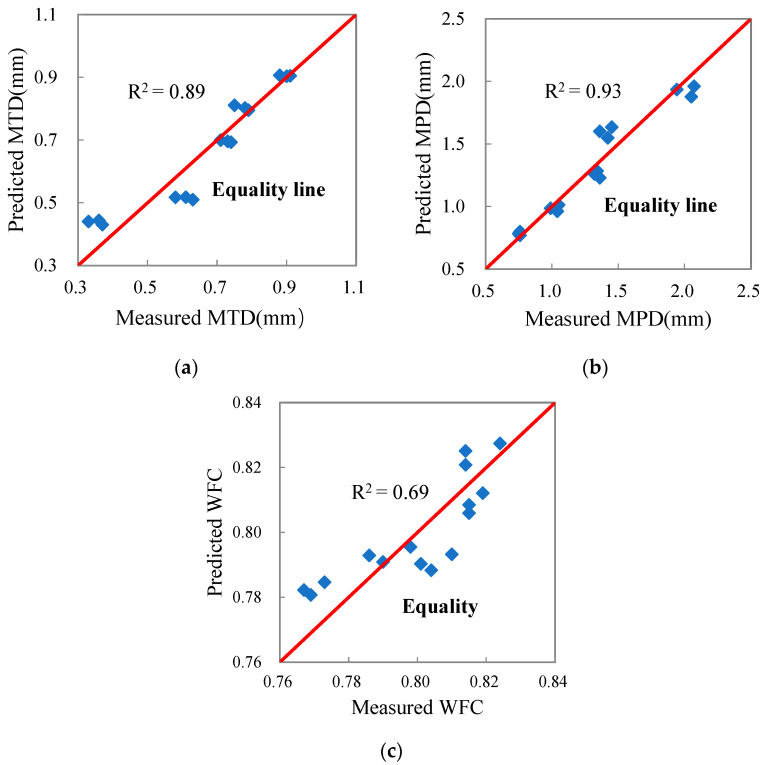
Predicted pavement surface property parameters and measured pavement surface property parameters: (**a**) MTD, (**b**) MPD, (**c**) WFC.

Figure 12 shows the correlation between the predicted pavement surface property evaluation indices calculated by the prediction models and the evaluation indices of pavement surface property measured by the sand patch method, 2D-ITAM and WFT. It can be seen that the data points are basically concentrated around the 45° equality line and the correlation coefficients are high.

## 6. Conclusions

This paper proposes three mathematical models to predict the surface properties of the asphalt pavements (Equations (9)–(11)). The models establish the correlation between aggregate geometrical properties and the asphalt pavement surface properties, which is of great significance to realize the optimization design of asphalt mixture based on pavement surface characteristics. The major findings of this study are summarized as follows:

(1) The Aggregate Geometric Characteristic Evaluation System (i.e., AGCES) developed based on 2-dimensional digital image processing techniques can characterize the form property, angularity characteristics, and surface texture of aggregate particles.
(2) Aggregate with distinct edges and corners and deeper surface texture improves pavement surface roughness, which is manifested as the increase in MTD, MPD and WFC. The form property of aggregate has no significant effect on the surface texture of asphalt pavement.
(3) The prediction models of pavement surface characteristics based on the angularity characteristics and surface texture of aggregate particles are developed according to the study on the influence of aggregate geometrical features on MTD, MPD and WFC. The coefficients of the mentioned models are calculated using Levenberg-Marquart and universal global optimization.

It should be noted that this paper only analyzes the correlations between aggregate geometry characteristics and pavement surface property. In fact, aggregate particle size, aggregate arrangement and aggregate gradation also directly affect pavement surface property, especially closely related to macro-texture. These are for subsequent research work.

## Figures and Tables

**Table 1:** Test program.

Number	Aggregate with Particle Size of 2.36 mm	Aggregate with Particle Size 2.36 mm	Aggregate Gradation	Asphalt Type
1	Limestone	Limestone	AC-13 coarse, AC-13 target and AC-13 fine	SBS modified asphalt
2		Round limestone		
3		Basalt		
4		Diabase		
5		Gneiss		

**Table 2:** Gradation of AC-13 asphalt mixture.

Gradation Type	16	13.2	9.5	4.75	2.36	1.18	0.6	0.3	0.15	0.075
AC-13 coarse	100	91	70	42	28	18	13	9	6	5
AC-13 target	100	96	78	44	33	23	17	11	9	6
AC-13 fine	100	99	83	64	46	35	25	18	14	7

**Table 3:** Influence of particle size on aggregate texture factor.

Aggregate Type	F	F_0.05_ (2477)
Limestone	921.496	3.015
Round limestone	781.542	
Basalt	804.315	
Diabase	764.862	
Gneiss	684.210	

**Table 4:** Influence of mineralogical properties on aggregate texture factor.

Aggregate Size (mm)	F	F_0.05_ (4795)
4.75	189.885	2.383
9.5	105.214	
13.2	129.643	

**Table 5:** Calculation method of aggregate geometrical features in the mixture.

Test Results	4.75 mm	9.5 mm	13.2 mm	Σ
Proportion of aggregate of a certain particle size in the gradation (%)	34	18	4	56
Proportion of aggregate of a certain particle size in coarse aggregate (%)	60.71	32.14	7.14	100
SI of aggregate	2.4312	2.224	2.0664	—
FF of aggregate	0.7893	0.7913	0.7945	—
AI of aggregate	1.0427	1.0340	1.0310	—
TF of aggregate	1.4386	0.5428	0.4211	—
Average SI of aggregates in the mixture (i.e., SIa)	2.338			
Average FF of aggregates in the mixture (i.e., FFa)	0.790			
Average AI of aggregates in the mixture (i.e., AIa)	1.039			
Average TF of aggregates in the mixture (i.e., TFa)	1.078			

**Table 6:** Geometric property results of aggregates in the mixture.

Aggregate Type	Gradation Type	SIa	FFa	AIa	TFa
Limestone	AC-13 coarse	2.302	0.791	1.038	0.971
	AC-13 target	2.338	0.790	1.039	1.078
	AC-13 fine	2.328	0.790	1.039	1.012
Round limestone	AC-13 coarse	2.221	0.808	1.017	0.849
	AC-13 target	2.246	0.808	1.016	0.938
	AC-13 fine	2.243	0.808	1.017	0.883
Basalt	AC-13 coarse	2.160	0.805	1.111	1.199
	AC-13 target	2.203	0.803	1.116	1.324
	AC-13 fine	2.184	0.803	1.116	1.244
Diabase	AC-13 coarse	2.252	0.797	1.080	0.934
	AC-13 target	2.290	0.796	1.082	1.033
	AC-13 fine	2.281	0.796	1.082	0.975
Gneiss	AC-13 coarse	2.149	0.811	1.144	1.455
	AC-13 target	2.197	0.808	1.148	1.591
	AC-13 fine	2.173	0.810	1.147	1.542

## Data Availability

Not applicable.

## References

[1] Arasan S., Yener E., Hattatoglu F., Hinislioglua S., Akbuluta S. (2011). Correlation between Shape of Aggregate and Mechanical Properties of Asphalt Concrete: Digital Image Processing Approach. Road Mater. Pavement Des..

[2] Bi Y., Guo F., Pei J., Zhang J., Li R. (2020). Correlation between geometric parameters of coarse aggregates and mixing rheological indices of asphalt mixture. Constr. Build. Mater..

[3] Sengoz B., Onsori A., Topal A. (2014). Effect of aggregate shape on the surface properties of flexible pavement. KSCE J. Civil. Eng..

[4] Zhou C., Zhang M., Li Y., Lu J., Chen J. (2019). Influence of particle shape on aggregate mixture’s performance: DEM results. Road Mater. Pavement Des..

[5] Kusumawardani D.M., Wong Y.D. (2020). The influence of aggregate shape properties on aggregate packing in porous asphalt mixture (PAM). Constr. Build. Mater..

[6] Kusumawardani D.M., Wong Y.D. (2021). Effect of aggregate shape properties on performance of porous asphalt mixture. J. Mater. Civ. Eng..

[7] Tutumluer E., Huang H., Bian X. (2012). Geogrid-aggregate interlock mechanism investigated through aggregate imaging-based discrete element modeling approach. Int. J. Geomech..

[8] Rao C., Tutumluer E. (2000). Determination of volume of aggregates: New image analysis approach. Transp. Res. Rec..

[9] Liu Y., Sun W., Nair H., Lane D.S., Wang L. (2016). Quantification of aggregate morphologic characteristics as related to mechanical properties of asphalt concrete with improved FTI system. J. Mater. Civ. Eng..

[10] Fletcher T., Chandan C., Masad E., Sivakumar K. (2003). Aggregate imaging system for characterizing the shape of fine and coarse aggregates. Transp. Res. Rec..

[11] Pan T., Tutumluer E. (2005). Imaging based evaluation of coarse aggregate size and shape properties affecting pavement performance. Advances in Pavement Engineering.

[12] Rao C., Tutumluer E., Kim I.T. (2002). Quantification of coarse aggregate angularity based on image analysis. Transp. Res. Rec..

[13] Topal A., Sengoz B. (2005). Determination of fine aggregate angularity in relation with the resistance to rutting of hot-mix asphalt. Constr. Build. Mater..

[14] Al-Rousan T., Masad E., Tutumluer E., Pan T. (2007). Evaluation of image analysis techniques for quantifying aggregate shape characteristics. Constr. Build. Mater..

[15] Bessa I.S., CasteloBranco V.T.F., Soares J.B., NogueiraNeto J.A. (2015). Aggregate shape properties and their influence on the behavior of hot-mix asphalt. J. Mater. Civil. Eng..

[16] Pei J., Bi Y., Zhang J., Li R., Liu G. (2016). Impacts of aggregate geometrical features on the rheological properties of asphalt mixtures during compaction and service stage. Constr. Build. Mater..

[17] Hassan H.M.Z., Wu K., Huang W., Chen S., Zhang Q., Xie J., Cai X. (2021). Study on the influence of aggregate strength and shape on the performance of asphalt mixture. Constr. Build. Mater..

[18] Usman T., Fu L., Miranda-Moreno L.F. (2010). Quantifying safety benefit of winter road maintenance: Accident frequency modeling. Accid. Anal. Prev..

[19] Mataei B., Zakeri H., Zahedi M., Nejad F.M. (2016). Pavement friction and skid resistance measurement methods: A literature review. Open J. Civil. Eng..

[20] Huang X., Zheng B. (2019). Research status and progress for skid resistance performance of asphalt pavements. China J. Highw. Transp..

[21] Shah S.M.R., Abdullah M.E. Effect of aggregate shape on skid resistance of compacted hot mix asphalt (HMA). Proceedings of the International conference on computer and network technology.

[22] Crouch L., Gothard J., Head G., Goodwin W. (1995). Evaluation of textural retention of pavement surface aggregates. Transp. Res. Rec..

[23] Abdul-Malak M., Fowler D.W., Constantino A. (1996). Aggregate characteristics governing performance of seal coat highway overlays. Transp. Res. Rec..

[24] Unal H., Mimaroglu A. (2013). Evaluation of morphologic characteristics and mechanical performance of rockforce mineral fiber- and glass fiber-reinforced polyamide-6 composites. Sci. Eng. Compos. Mater..

[25] Chen D., Sefidmazgi N.R., Bahia H. (2015). Exploring the feasibility of evaluating asphalt pavement surface macro-texture using image-based texture analysis method. Road Mater. Pavement Des..

[26] Han S., Sun P., Fwa T.F. (2021). Relationships between internal structure and surface texture of asphalt mixtures. Road Mater. Pavement Des..

[27] Han S., Liu M., Fwa T.F. (2020). Testing for low-speed skid resistance of road pavements. Road Mater. Pavement Des..

[28] ASTM International (2000). ASTM D3398 Standard Test Method for Index of Aggregate Particle Shape and Texture.

